# RhoA promotes osteoclastogenesis and regulates bone remodeling through mTOR-NFATc1 signaling

**DOI:** 10.1186/s10020-023-00638-1

**Published:** 2023-04-05

**Authors:** Jirong Wang, Chengyun Xu, Jing Zhang, Yizhong Bao, Ying Tang, Xiaoling Lv, Bo Ma, Ximei Wu, Genxiang Mao

**Affiliations:** 1 Zhejiang Provincial Key Lab of Geriatrics, Department of Geriatrics, Zhejiang Hospital, 1229 Gudun Road, Hangzhou, 310030 China; 2 Department of Pharmacology, Zhejiang University School of Medicine, 866 Yuhangtang Road, Hangzhou, 310058 China

**Keywords:** RhoA, Osteoclastogenesis, NFATc1, mTOR, Osteoporosis

## Abstract

**Background:**

The cytoskeletal architecture of osteoclasts (OCs) and bone resorption activity must be appropriately controlled for proper bone remodeling, which is associated with osteoporosis. The RhoA protein of GTPase plays a regulatory role in cytoskeletal components and contributes to osteoclast adhesion, podosome positioning, and differentiation. Although osteoclast investigations have traditionally been performed by in vitro analysis, however, the results have been inconsistent, and the significance of RhoA in bone physiology and pathology is still unknown.

**Methods:**

We generated RhoA knockout mice by specifically deleting RhoA in the osteoclast lineage to understand more about RhoA’s involvement in bone remodeling. The function of RhoA in osteoclast differentiation and bone resorption and the mechanisms were assessed using bone marrow macrophages (BMMs) in vitro. The ovariectomized (OVX) mouse model was adopted to examine the pathological effect of RhoA in bone loss.

**Results:**

Conditional deletion of RhoA in the osteoclast lineage causes a severe osteopetrosis phenotype, which is attributable to a bone resorption suppression. Further mechanistic studies suggest that RhoA deficiency suppresses Akt-mTOR-NFATc1 signaling during osteoclast differentiation. Additionally, RhoA activation is consistently related to the significant enhancement the osteoclast activity, which culminates in the development of an osteoporotic bone phenotype. Furthermore, in mice, the absence of RhoA in osteoclast precursors prevented occurring OVX-induced bone loss.

**Conclusion:**

RhoA promoted osteoclast development via the Akt-mTOR-NFATc1 signaling pathway, resulting a osteoporosis phenotype, and that manipulating RhoA activity might be a therapeutic strategy for osteoporotic bone loss.

**Supplementary Information:**

The online version contains supplementary material available at 10.1186/s10020-023-00638-1.

## Introduction

Bone is a living organ constantly renewed, and is maintained by the dynamic balance between bone formation and bone resorption (Matsushita et al. 2020). Associated with osteolytic diseases such as postmenopausal osteoporosis or bone metastasis (Bi et al. 2017), osteoclasts are responsible for pathologically reduced bone density. Osteoclasts are multinucleated large cells generated from hematopoietic stem cells that have a significant potential to resorb bone from the bone marrow (Boyle et al. 2003). Osteoclastogenesis is a process with osteoclastic precursors cellular migration, contact, and fusion into mature osteoclasts, that eventually combined the extracellular and intracellular cytokines secretion (Sims and Martin 2020). Importantly, receptor activator of NF-κB (RANK) ligand (RANKL) and macrophage colony-stimulating factor (M-CSF) regulate osteoclastic differentiation and function (Rao et al. 2018). Diverse transcription factors, such as AP and NFATc1, are activated when M-CSF and RANKL bind to their respective receptors (cFms and RANK). These factors in turn trigger various downstream mitotic activated protein kinases (MAPK) and the NF-κB signaling cascade (Honma et al. 2021). Multinucleated osteoclasts attach to calcified tissue and resorb bone by forming a resorption cavity, leading to hydroxyapatite dissolution in an acidic environment (Strzelecka-Kiliszek et al. 2017).

Rho subfamily GTPases are considered to be key regulators of the actin cytoskeleton, characterized as RhoA, Rac1, and Cdc42 of Ras GTPases (Masri and Delon 2021). They are a molecular switch that is triggered by diverse receptor signals between the active state as guanosine diphosphate (GDP) and GTP-linked action (Crosas-Molist et al. 2022). Rac1 and Rac2, are involved in the organization of the cellular cytoskeleton of osteoclast. This cytoskeleton modulates the adhesion of osteoclasts to bone and their subsequent resorption (Razzouk et al. 1999; Wang et al. 2009, 2008). Deletion of Rac1 in osteoclast precursors resulted in significant increases in bone mineral density (BMD) without altering osteoclast numbers (Magalhaes et al. 2011). In contrast, Croke et al. demonstrated that double KO of Rac1 and Rac2 resulted in an osteoporotic phenotype, with more, bigger, and irregular osteoclasts. Nevertheless, Rac1 or Rac2 loss alone mice have no skeletal phenotype (Croke et al. 2011). As downstream signaling of RANKL, Cdc42 might interact with Par3, Par6, and atypical PKC (Meriane et al. 2000), stimulating the osteoclastogenesis, causing osteopetrotic phenotype in Cdc42 knockout mice (Ito et al. 2010).

Several in vitro investigations utilizing pharmacological inhibitors or dominant-negative mutants have been used to investigate the role of RhoA in osteoclasts. Unfortunately, the findings retrieved by both methodologies are opposed. As a result, two groups of investigations indicated that RhoA is critical for the development, mobility, and bone resorption of osteoclasts (Chellaiah et al. 2000; Ory et al. 2000). However, Destaing et al. suggested that inhibition of RhoA mediates podosome patterning through Rho-mDia2-HDAC6 microtubule acetylation (Palazzo et al. 2001; Destaing et al. 2005). Therefore, we speculated that RhoA play an important role in normal bone physiology and the pathophysiology of many bone diseases. Recent studies have shown that Fasudil and Y-27632, ROCK (Rho-associated coiled-coil kinase) inhibitors enhance osteoclast formation, thus resulting in accelerated bone healing in rats with calvarial defects (Nakata et al. 2020). Despite their biological importance, research on the involvement of RhoA in osteoclast biology has shown conflicting results. The current work explores the RhoA impact through in vitro and in vivo analysis of osteoclast through genetic mouse models. Our findings have demonstrated that the absence of RhoA in osteoclast lineage impaired osteoclast formation and activity, leading to an osteopetrosis phenotype in mice. In addition, the gain-of-function of RhoA decreased bone mass by enhancing the activity of osteoclast. We have also revealed that RhoA exerts its skeletal effects by regulating osteoclastogenesis via Akt-mTOR-NFATc1 signaling. Following these results, it appears that targeting RhoA may be a promising technique for osteoporosis and other bone-resorption disorders.

## Results

### RhoA silencing inhibits osteoclastogenesis

In osteoclasts, RhoA is important for podosome assembly and osteoclast motility, however, the role in osteoclastogenesis is unknown. First, we examined the activity of RhoA in bone marrow macrophages (BMMs), extensively employed as main osteoclast precursors. The Rho-GTP pull-down assays revealed that GTP-RhoA expression was significantly increased in BMMs when stimulated with M-CSF and RANKL (Additional file 1: Figure S1a). Then, in vitro studies were performed to explore the role of RhoA in osteoclast differentiation. We used lentiviral production of short hairpin RNA against RhoA (shRhoA) in BMMs to suppress RhoA expression. The qRT-PCR analysis of the knockdown efficiency in comparison to control cells showed that it was > 80% (Additional file 1: Figure S1b). TRAP staining showed that RhoA silencing significantly decreased the production of osteoclasts by approximately 50% (Additional file 1: Figure S1c). Furthermore, RANKL induced the expression of *c-Fos* and *NFATc1*, the osteoclast differentiation transcription factor, at 2 days post treatments, while RhoA knockdown negated the induction. Treatment with RANKL significantly increased the expression of *DC-Stamp*, *TRAP*, and *Ctsk* at 4 days, which was suppressed by RhoA silencing (Additional file 1: Figure S1d). These findings proposed that RhoA may have a significant role in osteoclastogenesis.

### *RhoA*-depletion increases bone mass and reduced osteoclast activity

Subsequently, we generated *RhoA* knockout mice by specifically deleting *RhoA* in the osteoclast lineage to understand more about RhoA’s involvement in bone remodeling. It was observed that osteoclast precursors are targeted by Lysozyme M, whereas RNAK-Cre acts early in the osteoclast development process. Mouse strains expressing Cre recombinase driven by the RANK promoter (*RANK-Cre* mice) and Lysozyme M promoter (*LysM-Cre* mice) were crossed with *RhoA*^*f/f*^ mice. The subsequent progeny were bred with *RhoA*^*f/f*^ mice to produce *LysM-Cre; RhoA*^*f/f*^ (*LCre; RhoA*^*f/f*^) mice or *RANK-Cre; RhoA*^*f/f*^ (*RCre; RhoA*^*f/f*^) mice, depending on their genetic background.

**Fig. 1:**
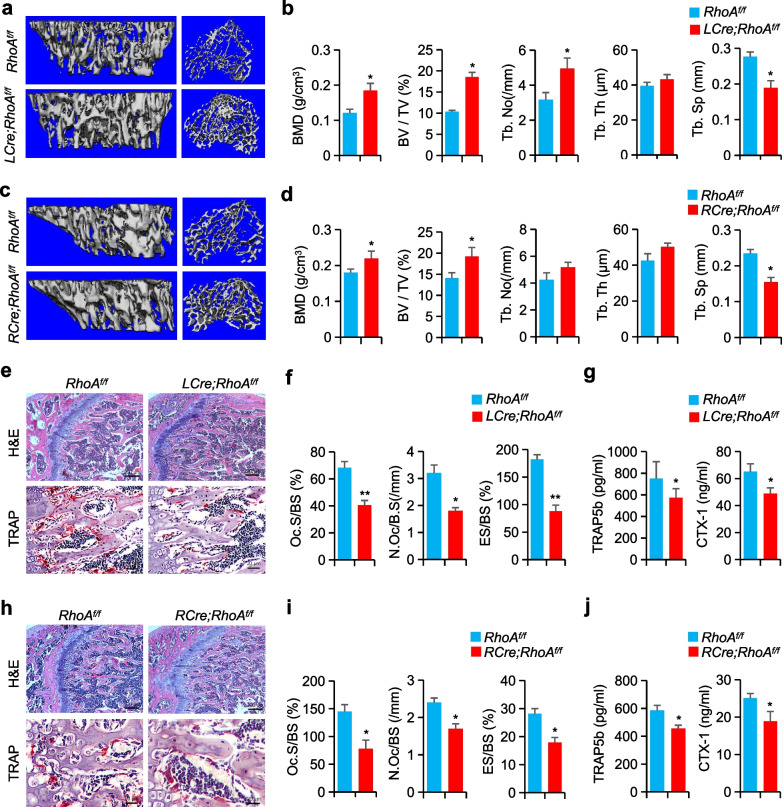
Osteosclerosis phenotype in *RhoA*-CKO mice. **a, c** Tibias of 3-month-old *RhoA*^*f/f*^ and *RhoA* CKO mice were imaged using a Micro-CT scanner. **b, d** The parameters of tibias growth plate trabecular bone. n = 6, BMD, bone mineral density; BV/TV, bone volume as a fraction of total bone volume; Tb.Th, trabecular thickness; Tb.No, trabecular number; Tb.Sp, trabecular separation. **e, h** TRAP and H&E staining images of mice femurs. Scale bars, 200 µm (H&E) and 20 µm (TRAP). **f, i** The parameters of femur osteoclasts. n = 6, ES/BS, eroded surface per bone surface; Oc.S/BS, osteoclast surface per bone surface; and N.Oc/BS, osteoclast number per bone surface. **g, j** Serum TRAP5b and CTX-1 levels, n = 6. Mean ± s.d., *P < 0.05, **P < 0.01, Student’s t-test

Bone marrows were isolated from *LCre; RhoA*^*f/f*^ or *RCre; RhoA*^*f/f*^ mice for detection of knockout efficiency. Results of qRT-PCR and western blotting showed very low expression of RhoA in the conditional knockout mice, respectively, compared with *RhoA*^*f/f*^ mice (Additional file 1: Figure S2a, b). In contrast to their *RhoA*^*f/f*^ littermates, 3-month-old *LCre; RhoA*^*f/f*^ or *RCre; RhoA*^*f/f*^ mice show no substantial morphological differences. In *LCre;RhoA*^*f/f*^ mice, however, the high-bone-mass phenotype was observed in the long bone. When compared to *RhoA*^*f/f*^ littermates, *LCre; RhoA*^*f/f*^ mice had a 1.5-fold increase in bone mineral density (BMD), a 1.8-fold increase in the ratio of bone volume to tissue volume (BV/TV), a 1.6-fold increase in trabecular number (Tb.No), and 30% decrease in trabecular separation (Tb.Sp) as measured by Micro-CT analysis of the proximal tibia (Fig. 1a, b). Similarly, Micro-CT scans of the long bones of 3-month-old *RCre; RhoA*^*f/f*^ mice confirmed the significant high-bone-mass phenotype (Fig. 1c). At three months, the proximal tibial BMD was higher than littermate control, with increased BV/TV, Tb.No but not Tb.Th, and lower Tb.Sp (Fig. 1d). Moreover, the *LCre; RhoA*^*f/f*^ and *RCre; RhoA*^*f/f*^ mice had more trabecular femurs when observed after the H&E staining (Fig. 1e, h). In contrast, structural parameters of cortical bone were not affected (Additional file 1: Figure S2c-f).

The activity of TRAP was significantly reduced in the main spongiosa of *LCre; RhoA*^*f/f*^ and *RCre; RhoA*^*f/f*^ mice femurs compared to *RhoA*^*f/f*^ littermates, which is consistent with our finding of an increase in BMD (Fig. 1e, h). When compared to control mice, bone-morphometric research indicated that the osteoclast surface (Oc.S/BS), osteoclast number (N.Oc/BS), and eroded surface (ES/BS) of *LCre; RhoA*^*f/f*^ and *RCre; RhoA*^*f/f*^ mice were all shown to be significantly lower in the latter two strains (Fig. 1f, i). Additionally, *LCre; RhoA*^*f/f*^ and *RCre; RhoA*^*f/f*^ mice had considerably lower levels of acid phosphatase 5, tartrate-resistant (TRAP5b) and C-terminal telopeptide of type I collagen (CTX-I) in serum than in control mice (Fig. 1g, j). It appears from these findings that osteoclast inactivation was the primary cause of the elevated bone mass observed in *RhoA*-deficient animals.

### *RhoA* deficiency suppresses osteoclastogenesis in vitro

**Fig. 2:**
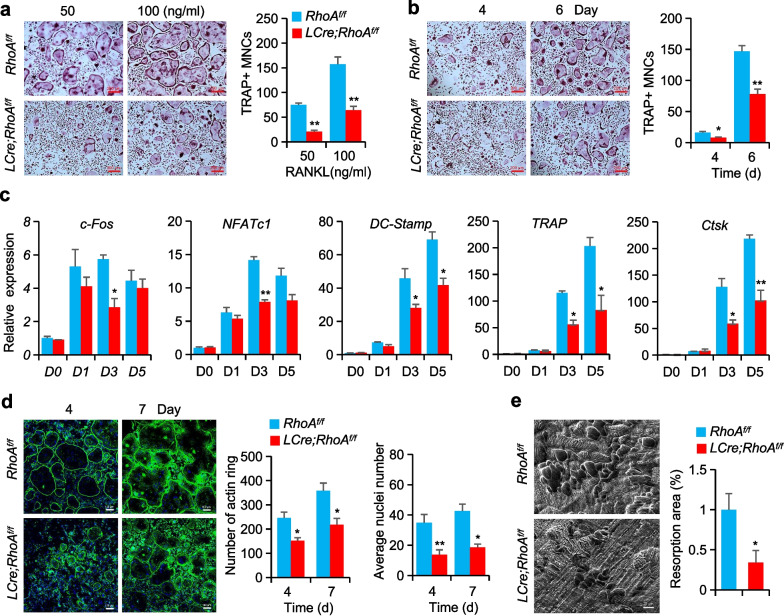
The absence of *RhoA* impairs the production and function of osteoclasts. **a, b** BMMs from *RhoA*^*f/f*^ and *LCre; RhoA*^*f/f*^ mice cultured with M-CSF (40 ng/ml) and varied dosages of RANKL (**a**) or specified periods with 50 ng/ml RANKL (**b**). The osteoclast numbers were counted (right). n = 6, Scale bars, 200 µm. **c** Expression of osteoclast-specific genes in *RhoA*^*f/f*^ and *LCre; RhoA*^*f/f*^ mice osteoclasts, n = 6. **d** Representative Phalloidin staining images of BMMs cultured with M-CSF and RANKL for 4 or 7 days, and quantification of F-actin ring per well and nuclei per osteoclast. n = 6, Scale bars, 100 µm. **e** SEM (scanning electron microscope) analysis and quantification of bone resorption area of bone slides, n = 6, Scale bars, 20 µm. Mean ± s.d., *P < 0.05, **P < 0.01, Student’s t-test or ANOVA analysis

Further, the role of RhoA in osteoclast development was investigated through in vitro analysis. When exposed to varied RANKL doses, *RhoA*-deficient cells generated much lower number and smaller-sized osteoclasts in BMMs compared to *RhoA*^*f/f*^ BMMs (Fig. 2a, Additional file 1: Figure S3a). Furthermore, the deficiency of *RhoA* slowed BMM differentiation, particularly at later stages (Fig. 2b). Gene expression study indicated that the expression of the osteoclast genes encoding *c-Fos*, *NFATc1*, *DC-stamp*, *TRAP*, and *Ctsk* was decreased in *RhoA*-deficient BMMs grown with M-CSF and RANKL for 3 or 5 days, but not in *RhoA*^*f/f*^ BMMs (Fig. 2c, Additional file 1: Figure S3b). Importantly, *DC-stamp* is one of the genes involved in osteoclast fusion, suggesting that RhoA controls osteoclast development by increasing the fusing of mononucleated osteoclasts into multinucleated osteoclasts.

The ability of osteoclasts to mediate effective bone resorption is indispensable to aid in the efficient organization of the cytoskeleton for the generation of a sealing zone. As a result, we investigated the effect of RhoA on the production of actin rings through in vitro studies. The osteoclasts from *LCre; RhoA*^*f/f*^ and *RCre; RhoA*^*f/f*^ mice displayed less actin ring structures than the osteoclasts from *RhoA*^*f/f*^ mice (Fig. 2d, Additional file 1: Figure S3c).

As a result of intensive investigation into the function of *RhoA* in osteoclastic bone resorption, we conducted in vitro bone resorption experiments as well as scanning electron microscopy (SEM) analysis of bone resorption pits. According to the obtained data, *LCre; RhoA*^*f/f*^ osteoclasts were shown to have a substantially smaller resorption area than the control osteoclasts (Fig. 2e), which had similar outcomes in *RCre; RhoA*^*f/f*^ mice (Additional file 1: Figure S3d).

Collectively, the in vitro results showed that *RhoA*-deficiency impairs RANKL-induced osteoclast differentiation and function.

### RhoA affects the mTOR signaling pathway

**Fig. 3:**
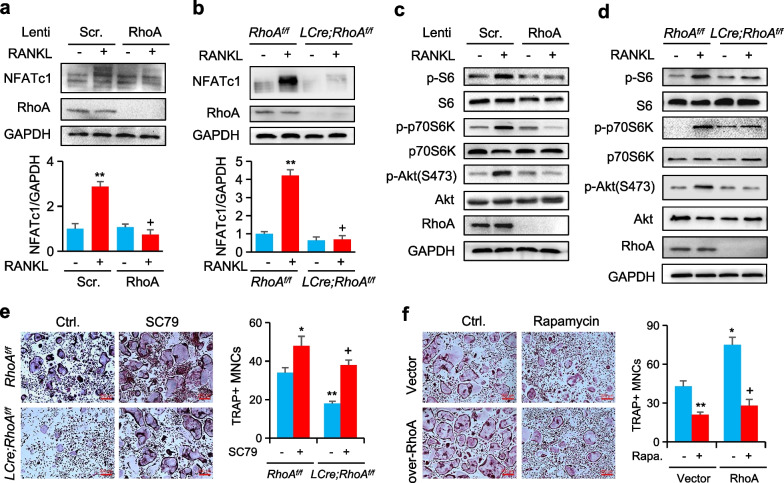
RhoA-mTOR-NFATc1signaling enhanced osteoclastogenesis. **a, b** Western blot of NFATc1 in RAW264.7 cells infected with shRhoA (**a**) and in BMMs from *RhoA*^*f/f*^ and *LCre; RhoA*^*f/f*^ mice and treated with RANKL for 24 h (**b**). **c** Western blot of S6, p70- S6K, and Akt phosphorylation in RAW264.7 cells infected by shRhoA and treated with RANKL for 15 min. **d** Western blot of S6, p70-S6K, and Akt phosphorylation in BMMs from *RhoA*^*f/f*^ and *LCre; RhoA*^*f/f*^ mice. **e** Representative TRAP staining images from *RhoA*^*f/f*^ and *LCre; RhoA*^*f/f*^ mice treated with or without SC79 (10 µM) respectively. **f** TRAP staining of BMMs infected by lenti-RhoA and treated with or without rapamycin (10 nM). n = 6, Scale bars, 200 µm. Mean ± s.d., *^,+^*P* < 0.05, **P < 0.01, Student’s t-test or ANOVA analysis

In osteoclast differentiation, the *NFATc1* transcription factor is the master transcription factor, controlling the expression of a variety of marker genes including *Ctsk* and *TRAP*, among several others. We first examined the NFATc1 expression by western blot analysis, the expression of NFATc1 induced by RANKL was downregulated in RAW264.7 cells infected with shRhoA (Fig. 3a). Similar results were obtained in *LCre; RhoA*^*f/f*^ cells (Fig. 3b). Numerous signaling cascades of RANKL-induced signaling (including stimulation of NFATc1 by p38, JNK, Erk, and NF-κB activation) work together to control osteoclast differentiation and the expression of NFATc1. The mammalian target of rapamycin (mTOR) is involved in anabolic metabolism in both osteoblasts and osteoclasts during the development of the skeleton. When it comes to modulating the activity of S6Kp70 ribosomal protein S6 kinase (S6K), it’s vital to remember that it is an enzyme that is controlled by PI3K effector and so plays a significant role in signaling downstream of mTORC1. Thus, our immunoblot analysis showed that Akt phosphorylation was induced by RANKL, but reduced both in shRhoA RAW264.7 cells and *LCre; RhoA*^*f/f*^ BMMs when compared to control (Fig. 3c, d). In addition, RANKL-induced phosphorylation of p70S6K and S6 were reduced in RhoA-deficiency BMMs relative to controls, similar results were observed in RAW264.7 cells (Fig. 3c, d). To determine whether mTOR functions downstream of RhoA signaling in osteoclasts, we then applied the Akt activator SC79 to analyze its function in osteoclastogenesis. TRAP staining revealed that SC79 increased the size of osteoclasts, whereas *RhoA*-deficient cells treated with SC79 generated osteoclasts at a normal level when compared to *RhoA*^*f/f*^ cells (Fig. 3e). Comparatively, rapamycin at 10 nM can normalize the hyper-activation of RhoA-overexpression osteoclasts (Fig. 3f).

Thus, the loss of *RhoA* results in the inhibition of the expression of NFATc1 by inhibiting the mTOR signaling.

### RANKL–RhoA–Rock-mTOR–NFATc1 pathway enhances the formation of osteoclasts

**Fig. 4:**
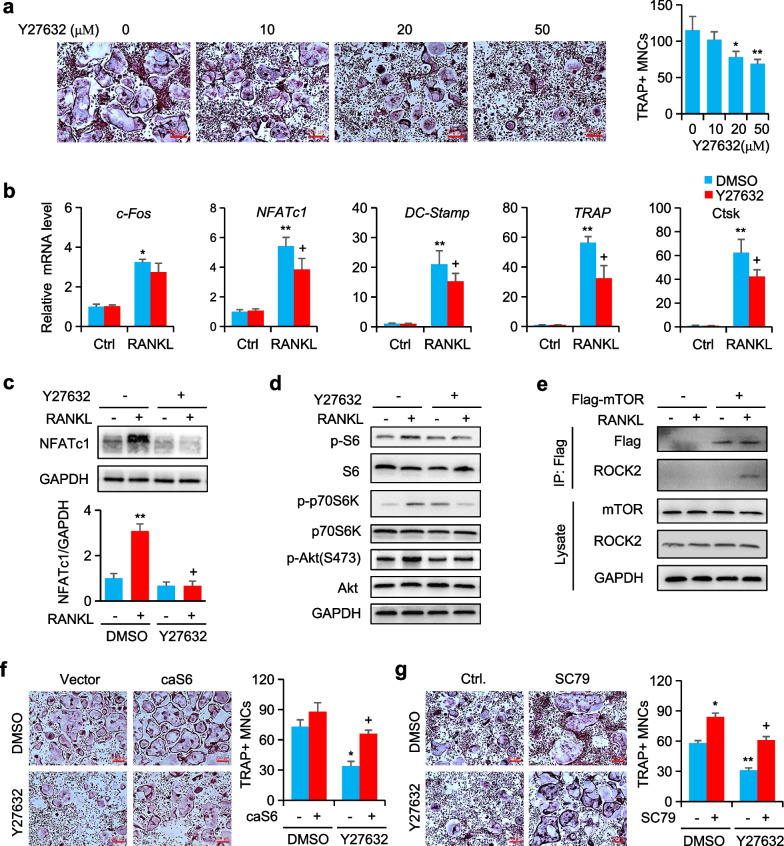
Rock enhanced the formation of osteoclasts by mTOR pathway. **a** Representative TRAP staining images of BMMs cultured with indicated doses of Y27632. **b** Expression of osteoclast-specific genes in BMMs cultured with 20 µM Y27632. **c** Western blot of NFATc1 in BMMs treated with Y27632 and RANKL. **d** Western blot of S6, p70-S6K, and Akt phosphorylation in BMMs treated with Y27632. **e** Co-IP of mTOR and Rock association in RAW264.7 cells with RANKL treated. **f** TRAP staining of BMMs infected with lenti-S6K1 and treated with or without Y27632. **g** Representative TRAP staining images of BMMs cultured with Y27632 and SC79 (10 µM). n = 6, Scale bars, 200 µm. Mean ± s.d., *^,+^*P* < 0.05, **P < 0.01, Student’s t-test or ANOVA analysis

Rock1/2 is one of the most extensively studied effectors of RhoA involved in a wide range of cell processes. To explore the role of Rock that acts similarly to *RhoA* deficiency in the regulation of osteoclastogenesis, we provided the control BMMs with a Rock2 inhibitor, Y27632. Dose-dependent inhibition of osteoclast production by Y27632 was observed, with a 70% reduction at 20 μM and a 60% reduction at 50 μM, respectively (Fig. 4a). The gene expression of osteoclast-specific genes was also decreased by Y27632 at 20 μM (Fig. 4b). Then, we examined the expression of transcription factor, western blot analysis showed that Y27632 decreased the RANKL-induced expression of NFATc1 (Fig. 4c). Similarly, RANKL-induced phosphorylation of p70S6K, S6 and Akt were reduced by Y27632 in BMMs (Fig. 4d). Then, by using a co-immunoprecipitation approach, we found that the Flag-tagged mTOR physically interact with Rock2 (Fig. 4e). Given the drastic decrease in S6K activation, we also observed that caS6K rescued the inhibition effect of Y27632 (Fig. 4f). We also analyzed the effects of SC79 dramatically reduced the inhibition of Y27632 (Fig. 4g). These results show that RhoA–Rock-mTOR signaling affects RANKL-induced osteoclastogenesis by promoting the expression of NFATc1.

### RhoA gain-of-function decreases bone mass and increases osteoclast activity

**Fig. 5:**
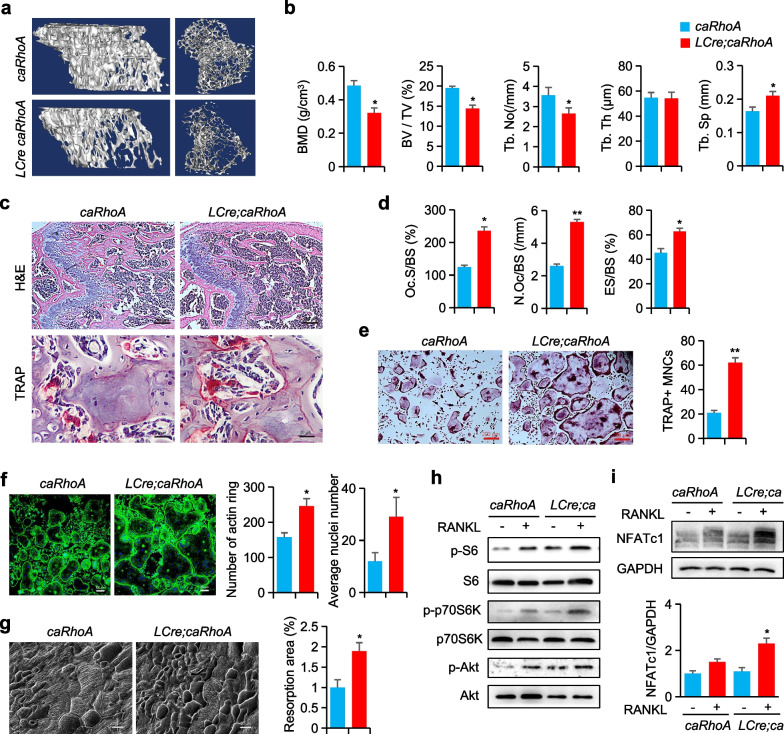
In osteoclast precursors, *RhoA* gain-of-function reduced the bone mass and increases osteoclast activity. **a** Representative Micro-CT images of 3-month-old *caRhoA* and *LCre; caRhoA* mice tibias. **b** The parameters of tibias growth plate trabecular bone, n = 6. **c** Representative H&E and TRAP staining images of mice femurs. Scale bars, 200 µm (H&E) and 20 µm (TRAP). **d** The parameters of femur osteoclasts, n = 6. **e** Representative TRAP staining images of BMMs from *caRhoA* and *LCre;caRhoA* mice. Scale bars, 200 µm. **f** Representative Phalloidin staining images of BMMs, and the quantification of F-actin ring per well and nuclei per osteoclast, n = 3, Scale bars, 100 µm. **g** SEM analysis and quantification of bone resorption area of bone slides, n = 6, Scale bars, 20 µm. **h** Western blot of S6, p70-S6K and Akt phosphorylation in BMMs from *caRhoA* and *LCre; caRhoA* mice and treated with RANKL for 15 min. **i** Western blot of NFATc1 in BMMs from *caRhoA* and *LCre; caRhoA* mice and treated with RANKL for 24 h. Mean ± s.d., *P < 0.05, **P < 0.01, Student’s t-test

The mice with the constitutively active form of *RhoA* (*caRhoA*, G14V) were then crossed with Cre-expressing osteoclasts to produce *LysM-Cre; caRhoA*^±^ (*LCre; caRhoA*) and *RANK-Cre; caRhoA*^±^ (*RCre; caRhoA*) mice. The results of the Micro-CT analysis revealed that *LCre; caRhoA* mice had a 30% decrease in BV/TV and Tb.No compared to control *caRhoA* mice. Additionally, Tb.Sp was increased by 40%, whereas BMD was decreased by 40% when compared to *caRhoA* littermates (Fig. 5a, b). Likely, in the *RCre; caRhoA* mice showed similar findings (Additional file 1: Figure S4a, b). When compared to *caRhoA* littermates, TRAP activity was significantly higher in *LCre;caRhoA* and *RCre;caRhoA* mice femurs at 3 months evidenced by elevated Oc.S/BS, N.Oc/BS, and ES/BS (Fig. 5c, d, and Additional file 1: Figure S4c, d). Furthermore, our findings revealed that constitutively active *RhoA* in the osteoclast lineage enhanced both the sensitivity of BMMs to RANKL and the size of osteoclasts (Fig. 5e, Additional file 1: Figure S4e). Consistently, the osteoclasts from LCre;caRhoA and *RCre;caRhoA* mice exhibited more actin ring structures and resorption area than the osteoclasts from control mice (Fig. 5f, g, and Additional file 1: Figure S4f, g). As predicted, *RhoA* activation also drastically enhanced the activity of mTOR signaling by increasing the phosphorylation of Akt, p70S6K, and S6 (Fig. 5h). Moreover, the expression of NFATc1 is increased during osteoclastogenesis in *LCre; caRhoA* BMMs compared to *caRhoA* cells (Fig. 5i).

### Loss of RhoA protects mice from pathological bone loss

**Fig. 6:**
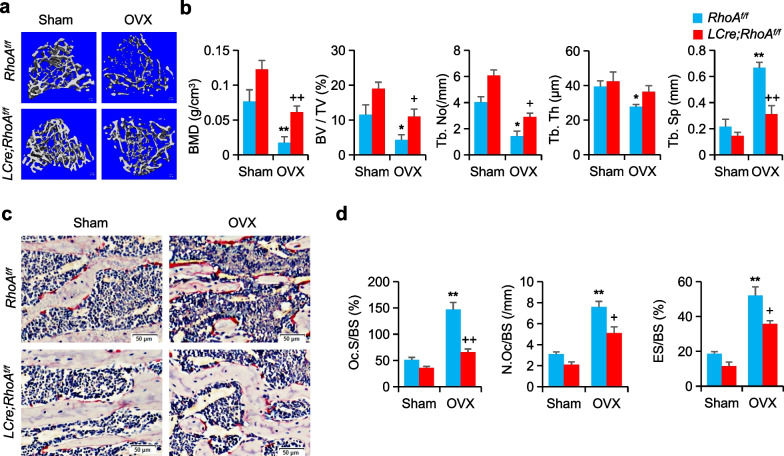
Loss of RhoA protects mice from pathological bone loss. **a** Representative Micro-CT images of *RhoA*^*f/f*^ and *LCre; RhoA*^*f/f*^ mice treated by sham or OVX. **b** The parameters of tibias growth plate trabecular bone, n = 6. **c** Representative TRAP staining images of mice femurs, Scale bars, 50 µm. **d** The parameters of femur osteoclasts, n = 6. Mean ± s.d., *^,+^*P* < 0.05, **^,++^*P* < 0.01, Student’s t-test or ANOVA analysis

Furthermore, we investigated the involvement of RhoA in an ovariectomized (OVX) animal model of osteoporosis to determine the possible therapeutic implications of RhoA-Rock. At 12 weeks after ovariectomy, the Micro-CT analysis of OVX therapy resulted in a reduced BMD in the *RhoA*^*f/f*^ mice tibial samples compared to sham control (Fig. 6a). In contrast, *LCre;RhoA*^*f/f*^ mice showed considerable resistance to bone loss, as demonstrated by increased BV/TV, Tb.No, and reduced Tb.Sp (Fig. 6b). TRAP staining further confirmed that loss of *RhoA* was associated with a reversal of OVX-induced decreases in TRAP-positive cell numbers in samples from these animals (Fig. 6c, d).

## Discussion

Osteoblast-stimulated osteoclastogenesis is an important component of bone remodeling. Bone deterioration is known to be caused by certain osteolytic disorders such as postmenopausal osteoporosis or bone metastasis. In the current study, we explore the role RhoA in osteoclastogenesis and resorption in vitro and in vivo studies through a variety of gain- and loss-of-function mouse genetic models. We here show that conditional knock-out RhoA in the osteoclast lineage restrains RANKL induced osteoclast formation and activity through inhibition of NFATc1 resulting in a severe osteopetrosis mouse phenotype. Furthermore, RhoA deficiency reduces mTOR signaling, which is involved in NFATc1 nuclei export, preventing osteoclast overactivation. OVX-induced osteoporosis in mice was prevented when RhoA was knocked out, indicating that this might be a potential therapy option for osteoporosis and other bone-resorption disorders in people.

In 1995, researchers presented the first evidence of RhoA involvement in the stability of podosomes. It has been observed that C3 inhibiting RhoA caused the ringed structure of podosomes to be disrupted (Ory et al. 2000; Zhang et al. 1995) and that C3 inhibiting RhoA impeded osteoclast polarization on apatite-coated slides (Saltel et al. 2004). Mice injected with constitutively active V14RhoA had podosomes vanish or be redistributed away from the cell's perimeter, in contrast to this finding (Chellaiah 2005). As a result of this apparent disparity, it is clear that the activity of RhoA must be tightly regulated during the production and patterning of podosomes. On the other hand, the inhibition of RhoA in the early stages of osteoclast differentiation accelerates podosome belt stabilization by regulating mDia2 and HDAC6 (Palazzo et al. 2001; Destaing et al. 2005). Consistent with the previous in vitro study, our data here confirmed that RhoA-deficiency impairs F-actin ring formation. Although the roles of RhoA in F-actin formation and stability were reported, its function in bone resorption and osteoclast differentiation has not been explored. Additionally, in osteoclast lineage, we found that RANKL-induces osteoclast production and bone resorption were reduced in the absence of RhoA.

According to prior research, Cdc42 deficiency resulted in osteopetrotic mice, whereas Cdc42 overexpression resulted in osteoporotic phenotype in mice. The further study found that Cdc42 influenced M-CSF-stimulated cyclinD expression and phosphorylation of Rb, as well as increased caspase 3 and Bim, which contributed to osteoclast proliferation and apoptosis rates (Ito et al. 2010). Osteoclasts were reduced in Rac1-deficient mice, which increased bone mass (Wang et al. 2008). In addition, the mice which was conditionally ablated Trio (Rac1 and Cdc42 exchange factor Triple functional domain) in monocytes, showed increased bone mass due to impaired bone resorption caused by osteoclasts (Gu et al. 2020). Additionally, we demonstrated in this work that mice with RhoA deletion in the osteoclast precursors and early stage of osteoclast development had a high bone mass phenotype, increased BV/TV and Tb.No, and reduced Tb.Sp, compared to wild-type mice. It was shown that RhoA activation in osteoclast lineage resulted in a considerable loss in bone mass, which we investigated further. We previously found that Wnt/catenin signaling is activated when RhoA is depleted in pre-osteoblasts, and Rock inhibition antagonizes bone loss associated with ageing (Shi et al. 2021), which is consistent with our current findings of increased bone mass in RhoA mice.

Previous research has shown that rapamycin suppression of mTORC1 promotes osteoclast differentiation from RAW264.7 macrophages (Shui et al. 2002), as well as enhanced bone remodeling and bone loss in healthy male rats (Rubert et al. 2015). Dai et al. conclude that deletion Raptor with Cathepsin K-Cre (Ctsk-Cre) limiting osteoclastogenesis and enhances bone mass (Dai et al. 2017). These outcomes are persistent with that of previous research demonstrating that mTORC1 stimulates osteoclast precursor growth in response to M-CSF activation (Glantschnig et al. 2003). Zhang et al. used LyzM-Cre to knock out Raptor or Tsc1 to inhibit or promote mTORC1 signaling and found that Raptor-deficient mice had osteopenia and increased osteoclastogenesis (Zhang et al. 2017). In another study, using LyzM-Cre and Vav1-iCre, Huynh et al. discovered that mTORC1 works as a dual regulator to govern the proliferation-to-differentiation transition during osteoclastogenesis, which is necessary for precursor proliferation but must be downregulated for differentiation (Huynh and Wan 2018). We found that in RhoA-deficient BMMs, RANKL-induced phosphorylation of p70S6K and S6 was decreased, which is per prior results on the role of mTORC1 in osteoclastogenesis. Most importantly, we found that the mTOR physically interact with Rock2 to regulate osteoclast differentiation.

For osteoclast differentiation, NFATc1 is both a necessary and sufficient transcription factor. Significantly, the embryonic stem cell deficiency of NFATc1 deforms the ability of osteoclasts differentiation. However, when the gene is overexpressed in osteoclast precursors in the absence of RANKL they differentiate into mature cells. Whereas, when the gene is targeted in hematopoietic cells in mice, bone mass is increased resulting in a significant decrease of osteoclasts (Takayanagi et al. 2002; Aliprantis et al. 2008). Therefore, a greater knowledge of NFATc1 regulation in osteoclasts will be critical for the understanding of bone disorders and the development of novel anti-resorptive therapeutics (Matsuo et al. 2004). Several signaling have been reported in regulation NFATc1, such as NF-κB, MAPK, and interferon regulatory factor-8 (IRF-8), recent studies have also uncovered negative regulations of NFATc1 through post-translational modifications and post-transcriptional regulations, such as ubiquitination, methylation, deacetylation (Kim et al. 2010; Nakamura et al. 2017). Following these findings, Zhang et al. concluded that mTORC1 reduces NF-κB/NFATc1 signaling, which in turn prevents osteoclast differentiation (Zhang et al. 2017). As established by Huynh et al., the protein mobility of NFATc1 decreases when it is activated by RANKL and dephosphorylated by calcineurin respectively (Huynh and Wan 2018). Our data showed that the expression of NFATc1 triggered by RANKL was downregulated in RAW264.7 cells infected with shRhoA, which was also evident for LCre; RhoA^f/f^ BMMs. Therefore, our results showed that RhoA controls osteoclastogenesis through Rock-mTOR-NFATc1 cascade.

Current Anti-resorptive medicines currently on the market are effective but not optimal. Bisphosphonates have a long-term detrimental impact on bone remodeling, which is the main issue. One of Denosumab’s adverse effects is calcium homeostasis imbalance and hypocalcemia (Reid and Billington 2022). To preserve normal bone homeostasis, it is preferable to use agents that restrict bone resorption by blocking the late development of osteoclasts without disrupting normal bone remodelling (Wu et al. 2017). Loss of RhoA, which inhibits osteoclast production and bone resorption, protected against OVX-induced bone loss, as demonstrated in the current study. The effects of RhoA/Rock in inflammatory disease models must be investigated further. Conclusively, inactivating RhoA might be a potential treatment for a variety of bone disease conditions.

## Conclusion

RhoA promoted osteoclast development via the Akt-mTOR-NFATc1 signaling pathway, resulting a osteoporosis phenotype, and that manipulating RhoA activity might be a therapeutic strategy for osteoporotic bone loss.

## Materials and methods

### Reagents

The fetal bovine serum (FBS) and α-MEM were provided by Gibco (California, USA). M-CSF and RANKLwere purchased from R&D (MN, USA). The tartrate-resistant acid phosphatase (TRAP) staining kit was purchased from Sigma-Aldrich (St Louis, MO, USA). HiPerFect Transfection Reagent was purchased from Qiagen (GmbH, Hilden, Germany). SC-79 was obtained from MedChemExpress (New Jersey, USA). Trap5b and CTX-I Elisa kit were purchased from Elabscience (Wuhan, China). Phalloidin-iFluor 488 (ab176753) was purchased from Abcam (Cambridge, UK). Antibodies for NFATc1 (#64,602) was from Biolegend (San Diego, CA). S6 (#2217), phospho-S6 (#4858), p70S6K (#9202), phospho-p70S6K (#9204), Akt (#4685), phospho-Akt (#31957), mTOR (#2983) and Rock2 (#47012) antibodies were from Cell Signaling Technology (Danvers, MA, USA). RhoA (ab187027) and GAPDH (ab8245) antibodies were from Abcam (Cambridge, UK). Flag (AF0036) antibody was from Beyotime Biotechnology (Shanghai, China).

### Mouse strains

The *RhoA*^*flox/flox*^ (*RhoA*^*f/f*)^ mice were generated by the Nanjing Biomedical Research Institute of Nanjing University (Nanjing, China) using a CRISPR/Cas9-mediated genome-editing technology. The conditional *caRhoA* (G14V) knock-in mouse strain was generated by Cyagen Biosciences Inc. (Guangzhou, China). The *LysM-Cre* mice was purchased from Nanjing Biomedical Research Institute. Moreover, Dr. Anqin donated the *RANK-Cre* mice (Shanghai Jiao Tong University School of Medicine, China). *LysM-Cre; RhoA*^*f/*+^ and *RANK-Cre; RhoA*^*f/*+^ males were crossed with *RhoA*^*f/f*^ females to produce *RhoA* deletion in osteoclastic lineage mice. Furthermore, *LysM-Cre* or *RANK-Cre* males were mated with *caRhoA* females to produce mice with *RhoA* activation in macrophages and osteoclasts. Throughout all of the studies, the WT littermates (*RhoA*^*f/f*^ or *caRhoA*) were compared to the mice that were bred with *LysM-Cre; RhoA*^*f/f*^, *RANK-Cre; RhoA*^*f/f*^, *LysM-Cre; caRhoA*^±^ or *RANK-Cre; caRhoA*^±^. Each mouse was bred on a C57BL/6 lineage. All animals were housed and bred at the Zhejiang University Animal Care Facility according to the institutional guidelines for laboratory animals, and the protocol was approved by the Zhejiang University Institutional Animal Care and Use Committee.

### BMMs isolation and cell culture

Primary BMM was extracted from long bones of 4-week old mice. Briefly, bone marrow cells were flushed, collected and washed twice with α-MEM. Then the cells were cultured with complete α-MEM (10% FBS and 100 U/ml penicillin–streptomycin) in the presence of M-CSF (40 ng/ml) for 4 days. RAW264.7 cells were maintained in complete α-MEM supplemented with 10% FBS and 100 U/ml penicillin–streptomycin. All the cells were maintained in a humidified incubator containing 5% CO_2_ at 37 °C.

### In vitro assessment of osteoclast formation

BMMs from mouse femoral bone marrow were obtained. Additionally, a 10-cm plate with a mixture of α-MEM and M-CSF (40 ng/mL) was utilized to cultivate cells until they achieved confluence. The BMMs were then cultured in α-MEM with 40 ng/mL M-CSF and 50 ng/mL RANKL for 7 days before being transferred for the next step. The RAW264.7 cells were cultured with α-MEM supplemented with 50 ng/ml RNAKL for 4 days. The TRAP activity of mature osteoclasts was assessed by staining and counting TRAP + MNCs per well in a 96-well plate.

### Quantitative reverse transcription-PCR (qRT-PCR) assay

Total RNA from the cells was collected using an RNA extraction kit (Takara Biotechnology Co., Ltd., Dalian, China) after cell treatment. Subsequently, using a ReverTra Ace qPCR RT Kit (TOYOBO, Osaka, Japan), 500 ng of RNA per sample was reverse transcribed results obtained from the qPCR (TOYOBO, Osaka, Japan).

The relative amounts of the mRNA levels of target genes were normalized to the GAPDH levels, respectively, and the relative difference in mRNA levels was calculated by 2^−ΔΔCt^ method, carried out using SYBR Green Realtime PCR Master Mix -Plus- (TOYOBO).

### Western blotting

BMMs or RAW264.7 cells were starved for 6 h and then stimulated with RANKL or M-CSF at different periods throughout this time for phosphorylation study.

Cell lysates were prepared by lysing cells directly in RIPA buffer supplemented with protease and phosphatase inhibitor and further quantified using a BCA protein assay kit (Solarbio, Beijing, China). 50 μg of total protein was subjected to SDS-PAGE followed by a transfer onto PVDF membranes (Millipore, Billerica, MA, USA). Membranes were incubated with primary antibodies against NFATc1, phospho-S6, p70S6K, phospho-p70S6K, Akt, phospho-Akt, mTOR, RhoA, Rock2 and GAPDH, followed by incubation in secondary antibodies. Immunosignals were developed by using the Enhanced Chemiluminescence System. GAPDH was used as internal standards.

### Actin ring formation and resorption pit assays

Initially, the mixture of M-CSF (40 ng/mL) and RANKL (50 ng/mL) was used to treat BMMs for 5 days in 12-well plates (1 × 10^4^/well) cover with glass coverslips. Further, a PBS rinse was followed by fixation in 4% PFA for 30 min before being blocked and stained with Phalloidin-iFluor 488 and DAPI. Fluorescent staining was examined using a ZEISS LSM900 spectral detector system. Fluorescence images were collected using ZEN Blue Lite software and analyzed using Image J software. Three or six wells were randomly selected for each group for further analysis, the average number of F-actin ring cells (> 3 nuclei/cell) per well and nuclei per cell were calculated.

We seeded BMMs in 96-well plates with an 8 × 10^3^/well density on bone slices and stimulated them similarly with M-CSF and RANKL for 8 days. After that, the resorption pits were examined under the scanning electron microscope (SEM; FEI Nova Nano SEM450) and analyzed using Image J software.

### RhoA activation assay

BMMs treated with M-CSF for 2 days were starved for 6 h before being stimulated with RANKL and M-CSF for varying durations. The RhoA-GTP activity was determined using commercial kits (Rho Activation Assay Biochem Kit, Cytoskeleton, Inc.) following the manufacturer's instructions after cell lysates were extracted and treated.

### Immunoprecipitation analysis

RAW264.7 cells were transfected with vector or Flag-mTOR plasmids. After 48 h, cells were treated with RANKL for 6 h, and then cell lysates were prepared with RIPA buffer with protease-inhibitor cocktail. The supernatant was incubated with Flag antibody at 4 °C overnight, which was followed by protein A/G bead incubation for another 3 h at 4 °C. Immune complexes were subjected to western blot analysis using specific antibodies for Flag and Rock (Cell Signaling Technology).

### Micro-CT analysis

The 3D reconstruction of bone and its respective parameter measurement was performed using a Micro-CT technique (Micro-CT 40, Scanco Medical AG, Brüttisellen, Switzerland) after the dissection of murine tibiae from euthanized animals. 100 16-μm slices just below the growth plate were used to reconstruct each picture (threshold = 200).

### Histology

Femurs from 3-month-old mice were fixed in 10% neutral buffered formalin and decalcified in 10% EDTA for 4 weeks. For H&E and TRAP staining, samples were embedded in paraffin and sectioned into 5-μm-thick longitudinal slices. Examination of femurs histology was performed according to routine histology techniques.

### OVX-induced bone destruction mouse models

On 2-month-old female mice, either an OVX procedure or a sham procedure was carried out. Analysis of Micro-CT images and TRAP staining were performed on mice that had been collected 8 weeks OVX post-operation and preserved in 4% PFA.

### Statistical analysis

All data are expressed as means ± S.D. In vitro experiments were performed in triplicate, while in vivo tests were conducted with six mice per group. All statistical analyses were performed by using the SPSS statistical package (IBM, North Castle, NY). Student’s t test or one-way ANOVAs were used to compare all data. *P* < 0.05 and *P* < 0.01 were the significance thresholds in this study. Reproducible outcomes are presented from all repeated triplicate studies.

## Supplementary Information

**Additional file 1. Figure S1**. Knock down of RhoA inhibits osteoclastogenesis in RAW264.7 cells. (a) The effect of RANKL and M-CSF stimulation on RhoA activity in pre-osteoclasts. (b) The qRT-PCR analysis following scrambling small hairpin RNA (shScr.) and RhoA shRNA (shRhoA) knockdown, n = 3. (c) Representative TRAP staining images of shRhoA infected RAW264.7 cells treated by RANKL for 4 days, n = 6, Scale bars, 200 μm. (d) The expression levels of the osteoclast-specific gene in shRhoA infected RAW264.7 cells for indicated times carried out with qRT-PCR, n=3. Mean± s.d., ^+^,*P < 0.05, ^++^,**P < 0.01, Student’s t-test or ANOVA analysis.** Figure S2**. Micro-CT measurement of cortical bone. (a) The western blotting of RhoA in BMMs from LCre;RhoA^*f/f*^ and RCre;RhoA^*f/f*^ mice. (b) The qRT-PCR of RhoA in BMMs from LCre;RhoA^*f/f*^ and RCre;RhoA^*f/f*^ mice. (c, e) 3D Micro-CT reconstructions of the tibial midshaft region. (d, f) Micro-CT measurement of cortical bone structural parameters from the midshaft region. n=6, Tt.Ar, total cross-sectional area; Ct.Ar, cortical bone area; Ct.Th, cortical thickness. Mean± s.d., **P < 0.01, Student’s t-test.** Figure S3**. Loss of RhoA in osteoclast inhibited osteoclastgenesis. (a) Representative TRAP staining images of BMMs from RhoA^*f/f*^ and RCre;RhoA^*f/f*^ mice cultured with M-CSF and RANKL. The osteoclast numbers were counted (right). n=6, Scale bars, 100 µm. (b) Expression of osteoclast-specific genes in RhoA^*f/f*^ and RCre;RhoA^*f/f*^ mice osteoclasts, n=6. (c) Representative Phalloidin staining images of BMMs, and the quantification of F-actin ring per well and nuclei per osteoclast. n=6, Scale bars, 50 µm. (d) SEM analysis and quantification of bone resorption area of bone slides, n=6, Scale bars, 20 µm. Mean±s.d., *P< 0.05, Student’s t-test or ANOVA analysis.** Figure S4**. RhoA gain-of-function in the early stage of osteoclast differentiation enhanced osteoclast activity and decreases bone mass. (a) Representative Micro-CT images of 3-month-old* caRhoA* and* RCre;caRhoA* mice tibias. (b) The parameters of tibias growth plate trabecular bone, n=6. (c) Representative H&E and TRAP staining images of mice femurs. Scale bars, 200 µm (H&E) and 20 µm (TRAP). (d) The parameters of femur osteoclasts, n=6. (e) Representative TRAP staining images of BMMs from caRhoA and* RCre;caRhoA* mice. Scale bars, 200 µm. (f) SEM analysis and quantification of bone resorption area of bone slides, n=6, Scale bars, 20 µm. (g) Representative Phalloidin staining images and quantification of F-actin ring per well and nuclei per osteoclast, n=3, Scale bars, 500 µm. Mean±s.d., *P< 0.05, Student’s t-test.

## Data Availability

The data that support the findings of this study are available from the corresponding author upon reasonable request.
