# Correction: RhoA promotes osteoclastogenesis and regulates bone remodeling through mTOR-NFATc1 signaling

**DOI:** 10.1186/s10020-023-00699-2

**Published:** 2023-07-31

**Authors:** Jirong Wang, Chengyun Xu, Jing Zhang, Yizhong Bao, Ying Tang, Xiaoling Lv, Bo Ma, Ximei Wu, Genxiang Mao

**Affiliations:** 1 Zhejiang Provincial Key Lab of Geriatrics, Department of Geriatrics, Zhejiang Hospital, 1229 Gudun Road, Hangzhou, 310030 China; 2 Department of Pharmacology, Zhejiang University School of Medicine, 866 Yuhangtang Road, Hangzhou, 310058 China

**Correction: Molecular Medicine (2023) 29:49** **https://doi.org/10.1186/s10020-023-00638-1**

Following publication of the original article (Wang et al. 2023), we have been informed that in Fig. 5h, the western blot image of p70S6K was incorrect due to the mistaken images being inadvertently inserted during the assembly of Fig. 5h. The correct Fig. 5h is given below:

**Fig. 5:**
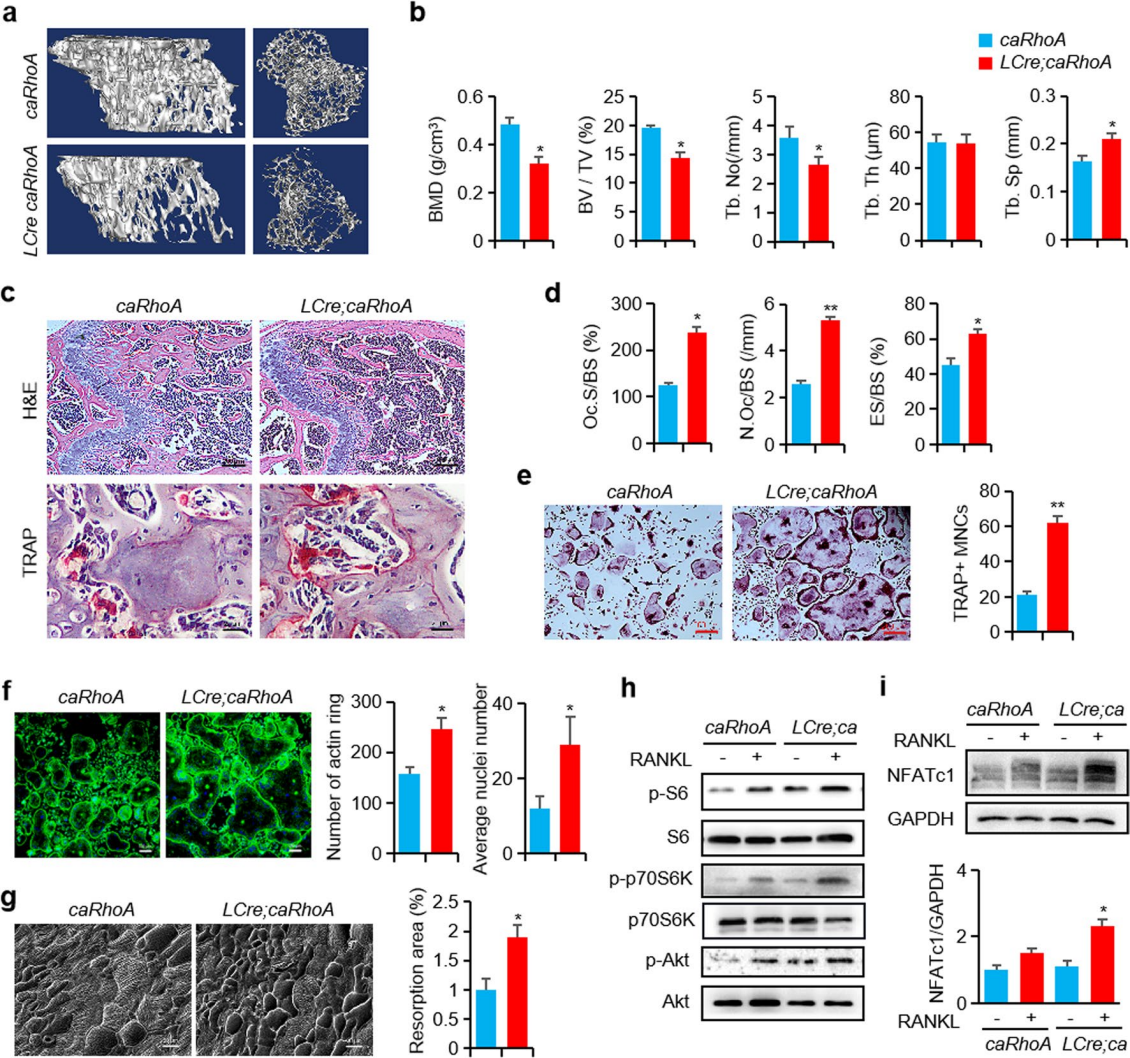
In osteoclast precursors, RhoA gain-of-function reduced the bone mass and increases osteoclast activity. **a** Representative Micro-CT images of 3-month-old caRhoA and LCre;caRhoA mice tibias. **b** The parameters of tibias growth plate trabecular bone, n = 6. **c** Representative H&E and TRAP staining images of mice femurs. Scale bars, 200 µm (H&E) and 20 µm (TRAP). **d** The parameters of femur osteoclasts, n = 6. **e** Representative TRAP staining images of BMMs from *caRhoA* and *LCre;caRhoA* mice. Scale bars, 200 µm. **f** Representative Phalloidin staining images of BMMs, and the quantification of F-actin ring per well and nuclei per osteoclast, n = 3, Scale bars, 100 µm. **g** SEM analysis and quantification of bone resorption area of bone slides, n = 6, Scale bars, 20 µm. **h** Western blot of S6, p70-S6K and Akt phosphorylation in BMMs from *caRhoA* and *LCre;caRhoA* mice and treated with RANKL for 15 min. **i** Western blot of NFATc1 in BMMs from *caRhoA* and *LCre;caRhoA* mice and treated with RANKL for 24 h. Mean ± s.d., *P < 0.05, **P < 0.01, Student’s t-test

Also, in Fig. S4, the image of H&E staining of RCre;caRhoA was incorrect. The correct Fig. S4 is given below: 

Additional file 1: Figure S4. *RhoA* gain-of-function in the early stage of osteoclast differentiation enhanced osteoclast activity and decreases bone mass. (a) Representative Micro-CT images of 3-month-old *caRhoA* and *RCre;caRhoA* mice tibias. (b) The parameters of tibias growth plate trabecular bone, n = 6. (c) Representative H&E and TRAP staining images of mice femurs. Scale bars, 200 µm (H&E) and 20 µm (TRAP). (d) The parameters of femur osteoclasts, n = 6. (e) Representative TRAP staining images of BMMs from *caRhoA* and *RCre;caRhoA* mice. Scale bars, 200 µm. (f) SEM analysis and quantification of bone resorption area of bone slides, n = 6, Scale bars, 20 µm. (g) Representative Phalloidin staining images and quantification of F-actin ring per well and nuclei per osteoclast, n = 3, Scale bars, 500 µm. Mean ± s.d., *P < 0.05, Student’s t-test.

The authors confirm all results, and conclusions of this article remain unchanged. The authors apologize for this error and any confusion it may have caused.
