# Supplementary material for: RhoA promotes osteoclastogenesis and regulates bone remodeling through mTOR-NFATc1 signaling
Source: Mol Med. 2023 Apr 5;29:49. doi: 10.1186/s10020-023-00638-1 (PMC10077675; doi:10.1186/s10020-023-00638-1)
Supplement: Supplementary file 1 — Additional file 1. Figure S1. Knock down of RhoA inhibits osteoclastogenesis in RAW264.7 cells. (a) The effect of RANKL and M-CSF stimulation on RhoA activity in pre-osteoclasts. (b) The qRT-PCR analysis following scrambling small hairpin RNA (shScr.) and RhoA shRNA (shRhoA) knockdown, n = 3. (c) Representative TRAP staining images of shRhoA infected RAW264.7 cells treated by RANKL for 4 days, n = 6, Scale bars, 200 μm. (d) The expression levels of the osteoclast-specific gene in shRhoA infected RAW264.7 cells for indicated times carried out with qRT-PCR, n=3. Mean± s.d., +,*P < 0.05, ++,**P < 0.01, Student’s t-test or ANOVA analysis. Figure S2. Micro-CT measurement of cortical bone. (a) The western blotting of RhoA in BMMs from LCre;RhoAf/f and RCre;RhoAf/f mice. (b) The qRT-PCR of RhoA in BMMs from LCre;RhoAf/f and RCre;RhoAf/f mice. (c, e) 3D Micro-CT reconstructions of the tibial midshaft region. (d, f) Micro-CT measurement of cortical bone structural parameters from the midshaft region. n=6, Tt.Ar, total cross-sectional area; Ct.Ar, cortical bone area; Ct.Th, cortical thickness. Mean± s.d., **P < 0.01, Student’s t-test. Figure S3. Loss of RhoA in osteoclast inhibited osteoclastgenesis. (a) Representative TRAP staining images of BMMs from RhoAf/f and RCre;RhoAf/f mice cultured with M-CSF and RANKL. The osteoclast numbers were counted (right). n=6, Scale bars, 100 µm. (b) Expression of osteoclast-specific genes in RhoAf/f and RCre;RhoAf/f mice osteoclasts, n=6. (c) Representative Phalloidin staining images of BMMs, and the quantification of F-actin ring per well and nuclei per osteoclast. n=6, Scale bars, 50 µm. (d) SEM analysis and quantification of bone resorption area of bone slides, n=6, Scale bars, 20 µm. Mean±s.d., *P< 0.05, Student’s t-test or ANOVA analysis. Figure S4. RhoA gain-of-function in the early stage of osteoclast differentiation enhanced osteoclast activity and decreases bone mass. (a) Representative Micro-CT images of 3-month-old caRhoA and RCr [file 10020_2023_638_MOESM1_ESM.pdf]

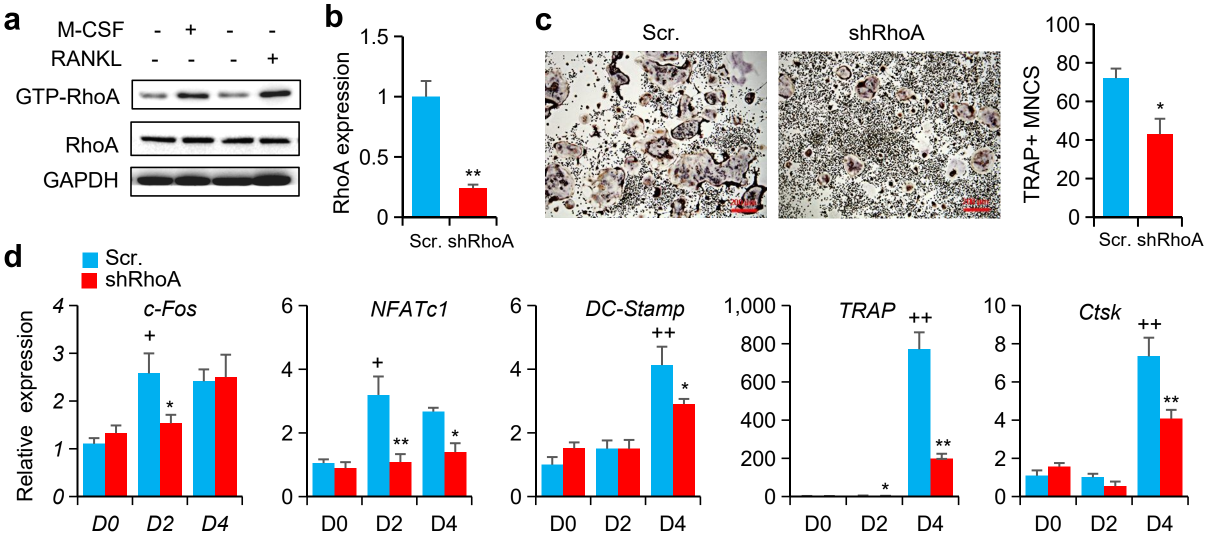

Supplementary Figure 1. Knock down of RhoA inhibits osteoclastogenesis in RAW264.7 cells.

(**a**) The effect of RANKL and M-CSF stimulation on RhoA activity in pre-osteoclasts. (**b**) The qRT-PCR analysis following scrambling small hairpin RNA (shScr.) and RhoA shRNA (shRhoA) knockdown, n = 3. (**c**) Representative TRAP staining images of shRhoA infected RAW264.7 cells treated by RANKL for 4 days, n = 6, Scale bars, 200 µm. (**d**) The expression levels of the osteoclast-specific gene in shRhoA infected RAW264.7 cells for indicated times carried out with RT-qPCR, n = 3. Mean± s.d., $^{+,*}P < 0.05$, $^{++,**}P < 0.01$, Student's t-test or ANOVA analysis.

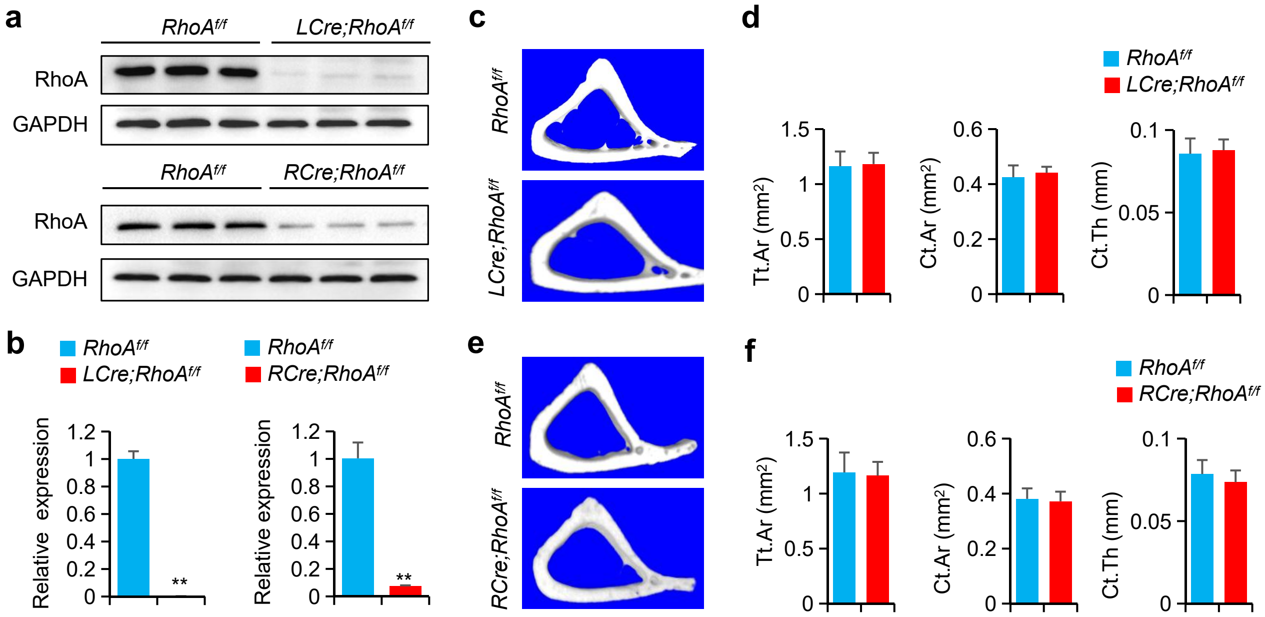

Supplementary Figure 2. Micro-CT measurement of cortical bone.

(**a**) The western blotting of RhoA in BMMs from *LCre;RhoA$^{f/f}$* and *RCre;RhoA$^{f/f}$* mice. (b) The qRT-PCR of RhoA in BMMs from *LCre;RhoA$^{f/f}$* and *RCre;RhoA$^{f/f}$* mice. (**c, e**) 3D Micro-CT reconstructions of the tibial midshaft region. (**d, f**) Micro-CT measurement of cortical bone structural parameters from the midshaft region. n=6, Tt.Ar, total cross-sectional area; Ct.Ar, cortical bone area; Ct.Th, cortical thickness. Mean± s.d., $^{**}P < 0.01$, Student's t-test.

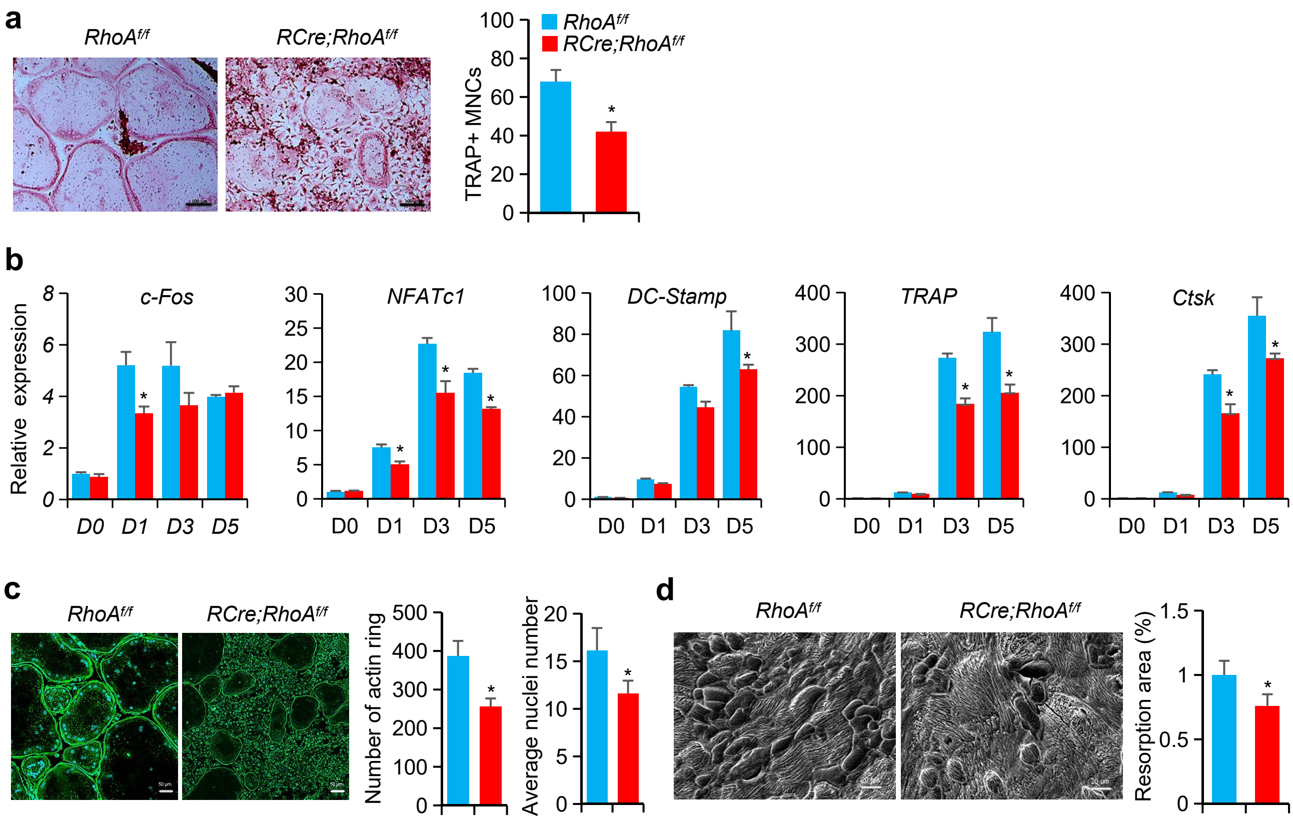

Supplementary Figure 3. Loss of *RhoA* in osteoclast inhibited osteoclastgenesis.

(**a**) Representative TRAP staining images of BMMs from *RhoA^f/f^* and *RCre;RhoA^f/f^* mice cultured with M-CSF and RANKL. The osteoclast numbers were counted (right). n=6, Scale bars, 100 µm. (**b**) Expression of osteoclast-specific genes in *RhoA^f/f^* and *RCre;RhoA^f/f^* mice osteoclasts, n=6. (**c**) Representative Phalloidin staining images of BMMs, and the quantification of F-actin ring per well and nuclei per osteoclast. n=6, Scale bars, 50 µm. (**d**) SEM analysis and quantification of bone resorption area of bone slides, n=6, Scale bars, 20 µm. Mean±s.d., $^{*}P< 0.05$, Student's t-test or ANOVA analysis.

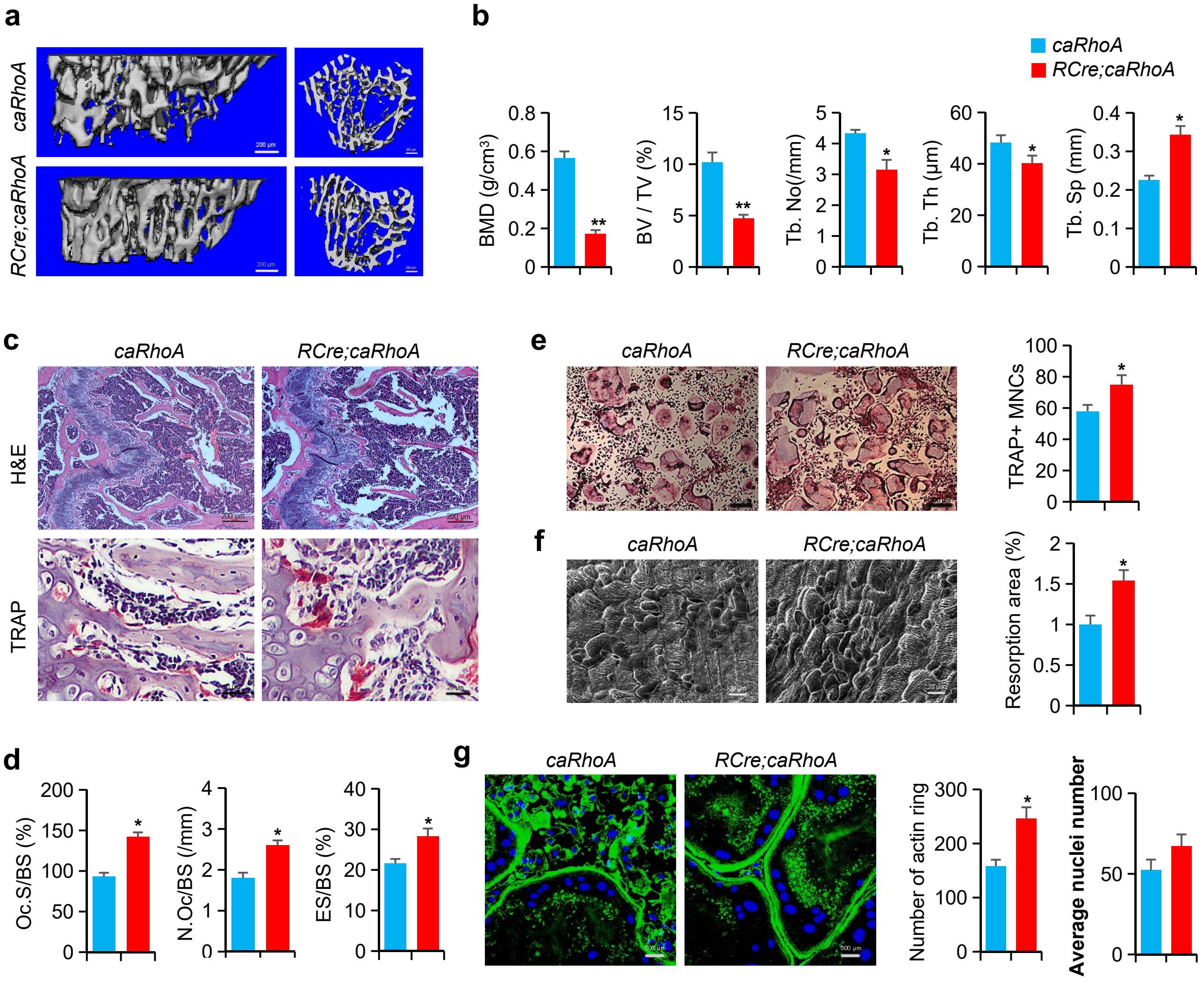

Supplementary Figure 4. *RhoA* gain-of-function in the early stage of osteoclast differentiation enhanced osteoclast activity and decreases bone mass.

(**a**) Representative Micro-CT images of 3-month-old *caRhoA* and *RCre;caRhoA* mice tibias. (**b**) The parameters of tibias growth plate trabecular bone, n=6. (**c**) Representative H&E and TRAP staining images of mice femurs. Scale bars, 200 μm (H&E) and 20 μm (TRAP). (**d**) The parameters of femur osteoclasts, n=6. (**e**) Representative TRAP staining images of BMMs from *caRhoA* and *RCre;caRhoA* mice. Scale bars, 200 μm. (**f**) SEM analysis and quantification of bone resorption area of bone slides, n=6, Scale bars, 20 μm. (**g**) Representative Phalloidin staining images and quantification of F-actin ring per well and nuclei per osteoclast, n=3, Scale bars, 500 μm. Mean±s.d., *$P< 0.05$, Student's t-test.
